# What Is the Minimum Clinically Important Change in Negative Symptoms of Schizophrenia? PANSS Based *Post-hoc* Analyses of a Phase III Clinical Trial

**DOI:** 10.3389/fpsyt.2022.816339

**Published:** 2022-04-25

**Authors:** Pál Czobor, Barbara Sebe, Károly Acsai, Ágota Barabássy, István Laszlovszky, György Németh, Toshi A. Furukawa, Stefan Leucht

**Affiliations:** ^1^Department of Psychiatry and Psychotherapy, Semmelweis University, Budapest, Hungary; ^2^Global Medical Division, Gedeon Richter Plc, Budapest, Hungary; ^3^Department of Health Promotion and Human Behavior, Kyoto University School of Public Health, Kyoto, Japan; ^4^Department of Psychiatry and Psychotherapy, School of Medicine, Technical University of Munich, Munich, Germany

**Keywords:** minimum clinically important difference, negative symptoms, schizophrenia, MCID, clinical trial, cariprazine

## Abstract

**Introduction:**

Minimum clinically important difference (MCID) is a measure that defines the minimum amount of change in an objective score of a clinical test that must be reached for that change to be clinically noticeable. We aimed to find the MCID for patients with predominantly negative symptoms of schizophrenia at its earliest occurrence.

**Methods:**

Data of a 26-week long, double-blind study with 454 patients [Positive and Negative Symptom Scale Negative Factor Score (PANSS-FSNS) ≥24, Positive and Negative Symptom Scale Positive Factor Score (PANSS-FSPS) ≤ 19] treated with cariprazine 4.5 mg/d or risperidone 4 mg/d were analyzed. The Clinical Global Impression—Improvement scale was used to quantify minimum improvement (CGI-I = 3) and no clinical change (CGI-I = 4) on the PANSS-FSNS, and the MCID was estimated with the following methods: as the mean PANSS-FSNS changes corresponding to the first instance of minimal improvement across all visits (MCID_1_); as the difference between the PANSS-FSNS change associated with the first instance and the PANSS-FSNS changes associated with the last recorded clinically unchanged status across all visits (MCID_2_); with the effect size approach (MCID_3_); as the Youden Index based cut-off value between no clinical change and minimal improvement (MCID_4_); as the relative likelihood of minimal improvement (MCID_5_).

**Results:**

The MCID_1_ and MCID_2_ resulted in, respectively, a 3.8-point (18.5%) and a 1.5-point (7.3%) decrease from baseline severity on the PANSS-FSNS. Greater values were required for the MCID at later evaluation times. The cut-off between minimum improvement and no clinical change defined by the Youden Index was a−3-point (15%) change in the PANSS-FSNS. The effect size approach indicated the 1.5-point difference between minimally improved and unchanged patients to be a medium effect (ES = 0.6).

**Conclusion:**

Applying different methods led to different results, ranging between 7.3 and 18.5% improvement from the baseline for the MCID at its earliest occurrence in patients with predominantly negative symptoms of schizophrenia.

## Introduction

The efficacy of various treatment interventions can be assessed in clinical trials by testing for statistical significance, yet a statistically significant change on a symptom scale score does not necessarily indicate a clinically relevant improvement (1). Thus, various approaches have been developed across different diseases to define the smallest beneficial effect for patients. One of the first attempts to obtain the slightest empirically observed, “clinically important” effects of intervention was published in 1989 by Jaeschke et al. (2), who defined the Minimum Clinically Important Difference (MCID) as “the smallest difference in score in the domain of interest which patients perceive as beneficial and which would mandate, in the absence of troublesome side effects and excessive cost, a change in the patient's management.” It is, therefore, a within-person, “before-after” change and conceptually distinct from minimum between-group differences that can be expected between two different treatments. The latter would be called the smallest worthwhile difference (SWD) (3). While the MCID is scale specific and assumes no substantial adverse effects or costs, the SWD represents the ratio of benefits and negative effects of two alternative treatments (4). Although other reports described the MCID with very similar names, such as the Minimal Clinically Important Difference (5, 6) Minimum Important Difference (7) or Minimal Important Change (6), the intended meaning is the same: MCID is a measure that defines the minimum amount of change in the objective score of a clinical test that must be reached for that change to be clinically noticeable.

Calculations of the MCID are usually divided into two groups: anchor- and distribution-based approaches. In anchor-based methods, an objective outcome measure of change is linked to a clinically meaningful external anchor, largely corresponding to patient perception (3, 6, 7) or in case of impaired insight, e.g., in dementia or schizophrenia, to clinical opinion (8–10). Distribution-based methods use statistical properties of study results, e.g., effect size or standard error of measurement, to calibrate the MCID (11–15).

In schizophrenia, the Positive and Negative Syndrome Scale (PANSS) measuring positive, negative, and general psychopathology, is the gold-standard instrument for assessing symptom severity (16). The clinical relevance of changes in the PANSS total score has been previously evaluated using the Clinical Global Impression (CGI) rating scale as an anchor (17, 18). It largely varies across different patient populations. Based on the CATIE study, where a very heterogeneous patient population was analyzed, a change of a 34% decrease on the PANSS total score was established as necessary to improve one category on the CGI-Severity scale (CGI-S) (18). For patients with florid positive symptoms, a 19–28% decrease in the PANSS total score was necessary to reach “minimal improvement” on the CGI-Improvement scale (CGI-I) (17). To specifically assess negative symptoms of schizophrenia, the PANSS factor score for negative symptoms (PANSS-FSNS) has been widely used in clinical trials (19). Leucht et al. found that minimal improvement corresponded to a change from baseline in PANSS-FSNS scores of −27 and −41%, as measured by the CGI-I and CGI-S (20).

Depending on the diagnostic criteria applied, negative symptoms of schizophrenia are present in 5–60% of patients with schizophrenia (21). These symptoms significantly affect a patient's quality of life as they limit functional recovery and are associated with poor functional outcomes (22). Patients with negative symptoms use more healthcare resources than patients with positive symptoms (such as: primary care, emergency care, laboratory and radiology tests, and prescription drugs), and their treatment is usually not simple, causing a clinical challenge for physicians. In contrast, positive symptoms have remarkably little association with real-life functioning and are easier to treat (23–25).

Finding the minimum clinically important change for negative symptoms may help physicians in better assessing treatment results as well as fostering the development of new instruments. In this paper, we further analyse the MCID in negative symptoms of schizophrenia, hypothesizing that as patients get better by taking their medication, more extensive changes in the PANSS-FSNS are needed to be considered clinically relevant. The previous estimation by Leucht et al. took all PANSS-FSNS changes associated with minimal improvement into account, regardless of their timepoint (20) meaning that the 27 and 41% improvement in the PANSS-FSNS associated with minimal clinical changes represent weighted averages from the first to the last instance of minimal improvement. Thus, those percentages may have overestimated the MCID in patients with predominantly negative symptoms of schizophrenia. In this work, we focus on the first instance of the MCID in patients with predominantly negative symptoms. Furthermore, to date, there is no consensus on the best method to calculate the MCID, and we apply both anchor- and distribution-based methods to get a more comprehensive picture.

## Methods

### Study Design

Data were analyzed from a large randomized, double-blind clinical trial treating patients with schizophrenia with predominantly negative symptoms; the study's methods and results have been previously published (26). The study was conducted at 66 study centers in 11 European countries from May 2013 to November 2014. The clinical study was approved by local independent ethics committees and was completed following clinical practice guidelines by the International Conference of Harmonization. All patients provided written informed consent. The study consisted of a 4-week lead-in period, a 26-week double-blind treatment period, and a 2-week safety follow-up with a total of 14 visits. The primary aim of the study was to assess the efficacy and safety of cariprazine treatment vs. risperidone treatment in primary, persistent, predominantly negative symptoms of schizophrenia. The primary efficacy outcome was the change from baseline in the PANSS-FSNS score at the end of the double-blind period (26 weeks). The secondary outcome parameter was change from the baseline on the Personal and Social Performance (PSP) scale. The study was well-controlled for secondary negative symptoms, assessing depression (Calgary Depression Scale for Schizophrenia), movement disorders (Simpson Angus Scale, Abnormal Involuntary Movement Scale, and Barnes Akathisia Rating Scale) and positive symptoms [PANSS factor score for positive symptoms (PANSS-FSPS)] throughout the study. Safety and tolerability were also assessed including adverse event reports, laboratory assessment, vital signs, and EEG. Patients were randomized to cariprazine 4.5 mg/d or risperidone 4 mg/d (1:1) with 2 weeks of up-titration (26).

### Patients

Male and female patients with schizophrenia and predominantly negative symptoms, between 18 and 65 years of age, who were diagnosed with schizophrenia (as defined by DSM-IV-TR) and an onset of illness ≥2 years prior, were included in the study. Patients also needed to be in stable condition for at least 6 months with no hospitalisations. For study inclusion, patients must have presented with predominantly negative symptoms for ≥6 months, a PANSS-FSNS ≥24, and a score ≥4 on at least 2 of the following 3 PANSS negative symptom items: blunted affect, passive/apathetic social withdrawal, and lack of spontaneity and flow of conversation. The presence of a current DSM-IV-TR axis I disorder other than schizophrenia, and an unstable condition (such as a hospital admission in the previous 6 months), a PANSS factor score for positive symptoms (PANSS-FSPS) >19, or a PANSS-FSPS score increase of ≥25% during study lead-in were grounds for study exclusion. Other clinical exclusion criteria included substance abuse/dependence, treatment with clozapine during the 12 months before the study, a history of non-response to an adequate trial of risperidone for a psychotic episode, or treatment with risperidone within 6 weeks of screening (26).

### MCID Analyses

The clinician-rated CGI-I scale was used to quantify minimum improvement (CGI-I = 3) and no clinical change (CGI-I = 4) on the PANSS-FSNS. To demonstrate any meaningful results by linking an objective scale measuring symptom severity to a subjective scale that estimates the clinical state correlation between the two scales must be demonstrated in the target population. This was done by Leucht et al., who performed the correlation analysis in the same population on which this work is based (20).

According to the definition of the MCID, a within-subject design was applied to estimate the difference between no clinical change and minimal improvement. Our observation was that PANSS-FSNS changes corresponding to minimal improvement get higher and higher over time, and thus, in order to capture the minimum clinically important difference, the MCID should be calculated on the basis of PANSS-FSNS changes associated with the earliest instance of minimal improvement. The MCID was estimated with the following methods.

#### Anchor-Based Methods

- MCID_1_: The mean PANSS-FSNS changes corresponding to the first instance of minimal improvement (CGI-I = 3) across all visits. In other words, MCID_1_ is based on the original definition by Jaeschke et al. (2) for MCID, as it represents a change from baseline in the original score units of the PANSS-FSNS scale.
- MCID_2_: The difference between the PANSS-FSNS change associated with the first instance of improvement (i.e., CGI-I = 3) and the PANSS-FSNS changes associated with the last recorded clinically unchanged status (CGI-I = 4) across all visits. Thus, MCID_2_ represents the mean score method of Redelmeier and Lorig (27) i.e., it shows the score difference of the “slightly better” group minus that of the “about the same” group (28). Accordingly, MCID_2_ is also expressed in the original scale units.

To test the statistical significance of the difference between unchanged and minimally improved PANSS-FSNS values, we applied a mixed model for repeated measures (MMRM) analysis with improvement and visit as fixed effects. The subject was used as random effect in the model. To avoid losing cases with minimal improvement at the first visit after baseline (Week 1), zero PANSS-FSNS change was imputed for the baseline visit (where no improvement can be present by definition).

#### Distribution-Based Methods

- MCID_3_: the effect size approach, based on the standardized response mean difference, a widely used distribution-based method to estimate the MCID, where MCID is the mean difference between the last unchanged (CGI-I = 4) and the first minimally improved (CGI-I = 3) PANSS-FSNS values divided by the pooled standard deviation (SD) of the two. It formally corresponds to the effect size calculation, making it possible to interpret the MCID in terms of the effect sizes (28).
- MCID_4_: dichotomous variable indexing minimal improvement (not obtained = 0, obtained = 1) based on the cut-off value between no clinical change (CGI-I = 4) and minimal improvement (CGI = 3). A logistic regression model, with CGI-I as dependent and PANSS-FSNS as independent variables as well as baseline PANSS-FSNS as a covariate, was fitted to the data. To examine the accuracy of predicting improvement based on the PANSS-FSNS change, a receiver operating characteristics (ROC) curve was derived. The strategy used in the ROC analysis was to maximize both sensitivity and specificity, and the MCID was estimated as a cut-off value corresponding to the maximal Youden's index (29, 30).
- MCID_5_ as expressed in terms of ratio of odds values (p/1-p) of being in improved vs. unchanged state at a certain degree of FSNS decrease. This estimation is based on the logistic regression model as above and expresses the strength of the predictive power of unity change in FSNS for clinical improvement.

## Results

The primary results of the study were previously published (26): change from baseline to week 26 in PANSS-FSNS was significantly greater with cariprazine than with risperidone [least squares mean difference (LSMD) −1.46, 95% CI −2.39 to −0.53; *p* = 0.0022; effect size = 0.31]. Also, for the secondary efficacy parameter, least squares mean change from baseline to endpoint in PSP total score, was greater for cariprazine than risperidone (LSMD 4.63, 2.71–6.56; *p* < 0.0001; statistical effect size = 0.48). In the parameters controlling for secondary negative symptoms, least squares mean changes from baseline for PANSS-FSPS, CDSS total score, and movement scales were small and similar for cariprazine and risperidone.

A total of 454 patients from the intent-to-treat population with at least one post-baseline PANSS assessment were pooled for this analysis from both the cariprazine and risperidone treatment groups; the mean factor score on the PANSS-FSNS at baseline was 27.6, with points decreasing to 19.0 points at week 26.

### By Visit Analyses

**Figure 1:**
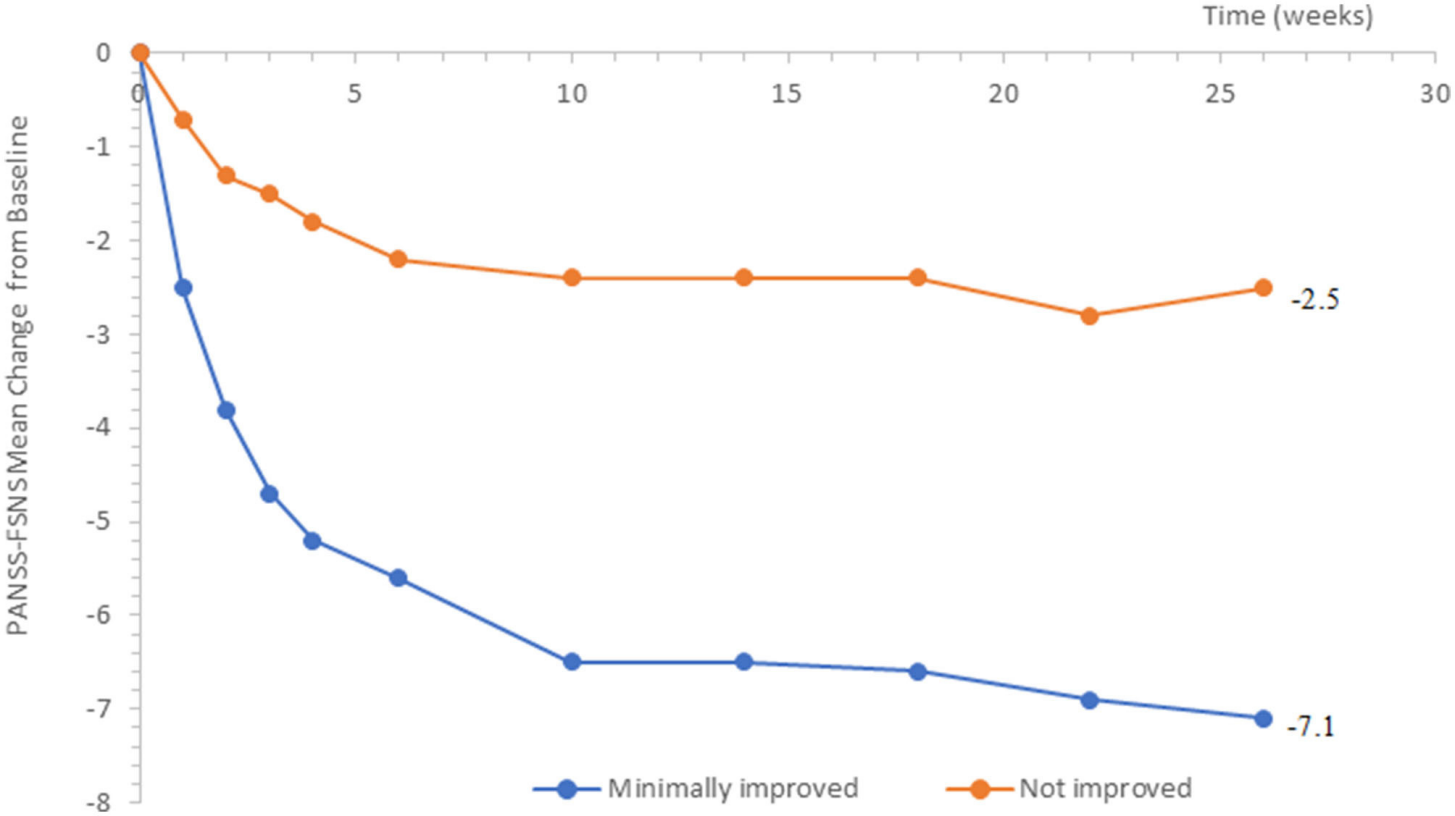
Change from baseline in PANSS-FSNS (Positive- and Negative Syndrome Scale—Factor Score for Negative Symptoms) as a function of minimal change vs. no change.

Minimal improvement on the clinical global impression scale (CGI-I = 3) was associated with PANSS-FSNS changes ranging from −2.5 points (Week 1) to −7.1 points (Week 26), consistent with our hypothesis of the MCID to be smaller at its earliest occurrence (Figure 1).

### Quantifying the MCID by Anchor-Based Methods

#### MCID_1 and 2_

The mean PANSS-FSNS change from baseline corresponding to the first occurrence of minimal improvement (CGI-I = 3) and the last recorded unchanged status across all visits were −3.8 and −2.3 points, respectively. The statistical analysis of the two arithmetic PANSS-FSNS means showed a significant difference (Table 1).

**Table 1:** Anchor based calculations of the MCID.

**Visit**	**Mean PANSS-FSNS change from baseline (*n*) at the 1st instance of minimal improvement [1]**	**Mean PANSS-FSNS change from baseline (*n*) at the last recorded unchanged status [2]**	**MCID_**1**_ [1]**	**MCID_**2**_ [1]-[2]**	**SD of [1] and [2]**	**LSMD (95% CI)**	***P*-value**
Overall	−3.8 (365)	−2.3	−3.8 (18.5%)	−1.5 (7.3%)	2.5	1.7 (1.1, 2.3)	<0.0001

*PANSS-FSNS, Positive and Negative Syndrome Scale-Factor Score for Negative Symptoms; n, number of events; CGI-I, Clinical Global Impression—Improvement; MCID_1_ and MCID_2_, Minimum clinically important differences according to the definitions in the text; SD, Standard deviation; LSMD, Least-square mean difference between [1] and [2], as estimated by MMRM, with corresponding 95% confidence limits. Please also note that for the computation of % changes from baseline the value of 7 was subtracted from the observed baseline severity (i.e., 27.6) since the minimum value of the PANSS-FSNS factor is 7 (i.e., the symptoms on all seven constituting items of the factor are rated as “Absent”)*.

### Quantifying the MCID by Distribution-Based Methods

#### MCID_3_

Based on the observed PANSS-FSNS changes the standardized effect size was determined according to the following formula: (PANSS-FSNS change from baseline at the first instance minimal improvement) − (Mean PANSS-FSNS Change from baseline at the last recorded unchanged status)/pooled SD. Our computation resulted in a standardized effect size for the improvement with a value of 0.6 [i.e., −3.8 – (−2.3)/2.5 = −1.5/ 2.5 = −0.6].

#### MCID_4_

**Figure 2:**
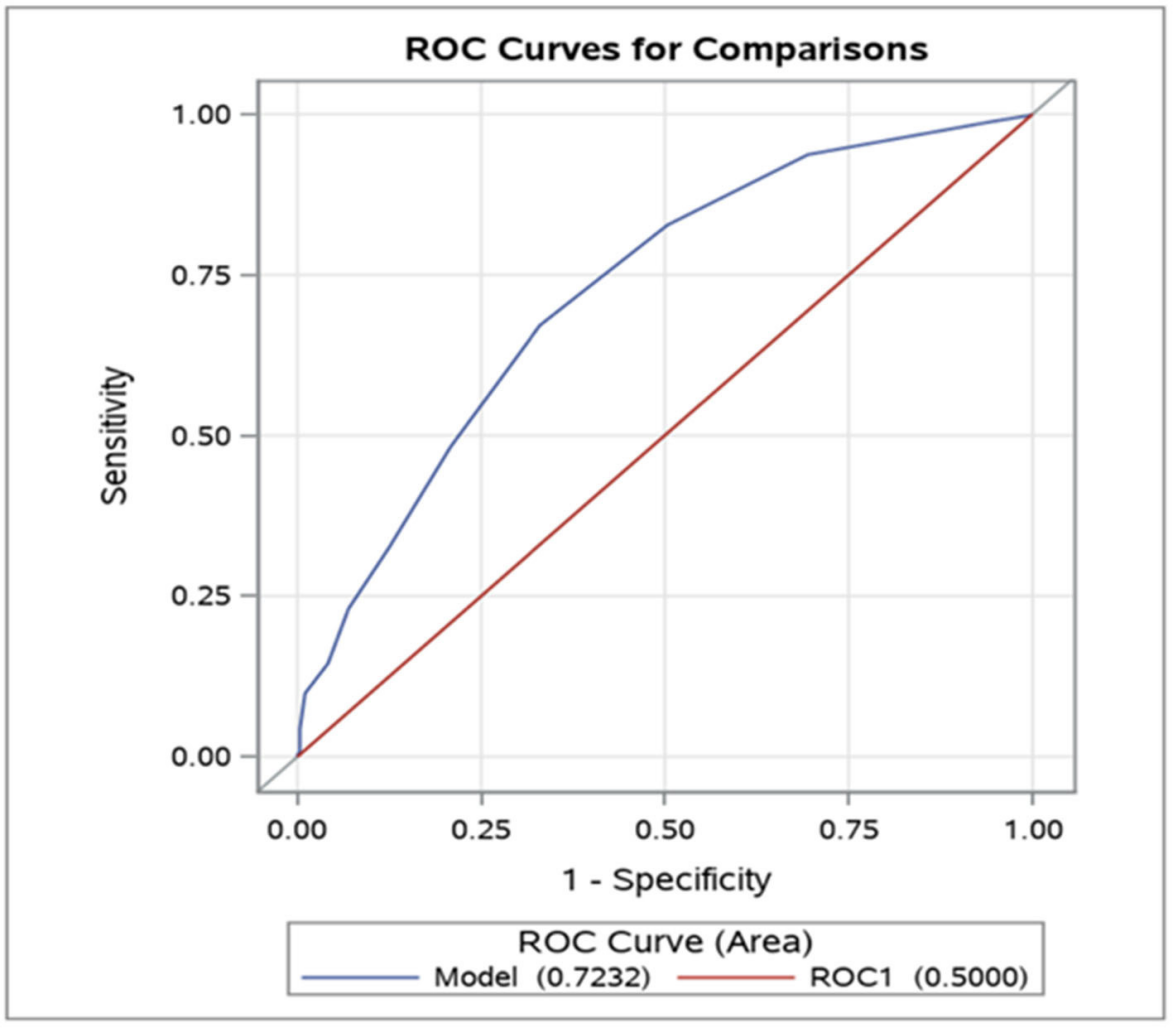
Receiver Operating Characteristic (ROC) curve: predictive accuracy of the PANSS-FSNS (Positive and Negative Syndrome Scale Factor Score for Negative Symptoms) scale for differentiating minimally improved vs. clinically unchanged status. The values on the vertical and horizontal axis, respectively, depict the sensitivity (“true positive rate”) and 1− specificity (“false positive rate”) values for the differentiation as a function of change from baseline in the PANSS-FSNS scale. The leftmost part of the ROC curve represents the highest empirically observed improvements in the sample as compared to baseline while the rightmost part represents no improvement (or even deterioration). Please note that the ROC curve for differentiating the minimally improved from the clinically unchanged status based on the PANSS-FSNS (ROC model, depicted in blue in the figure) significantly outperforms the random classification (ROC1 model, in red), with an area under the curve (AUC, labeled as “Area”) value of 0.7232 vs. 0.5000 (*p* < 0.0001).

**Figure 3:**
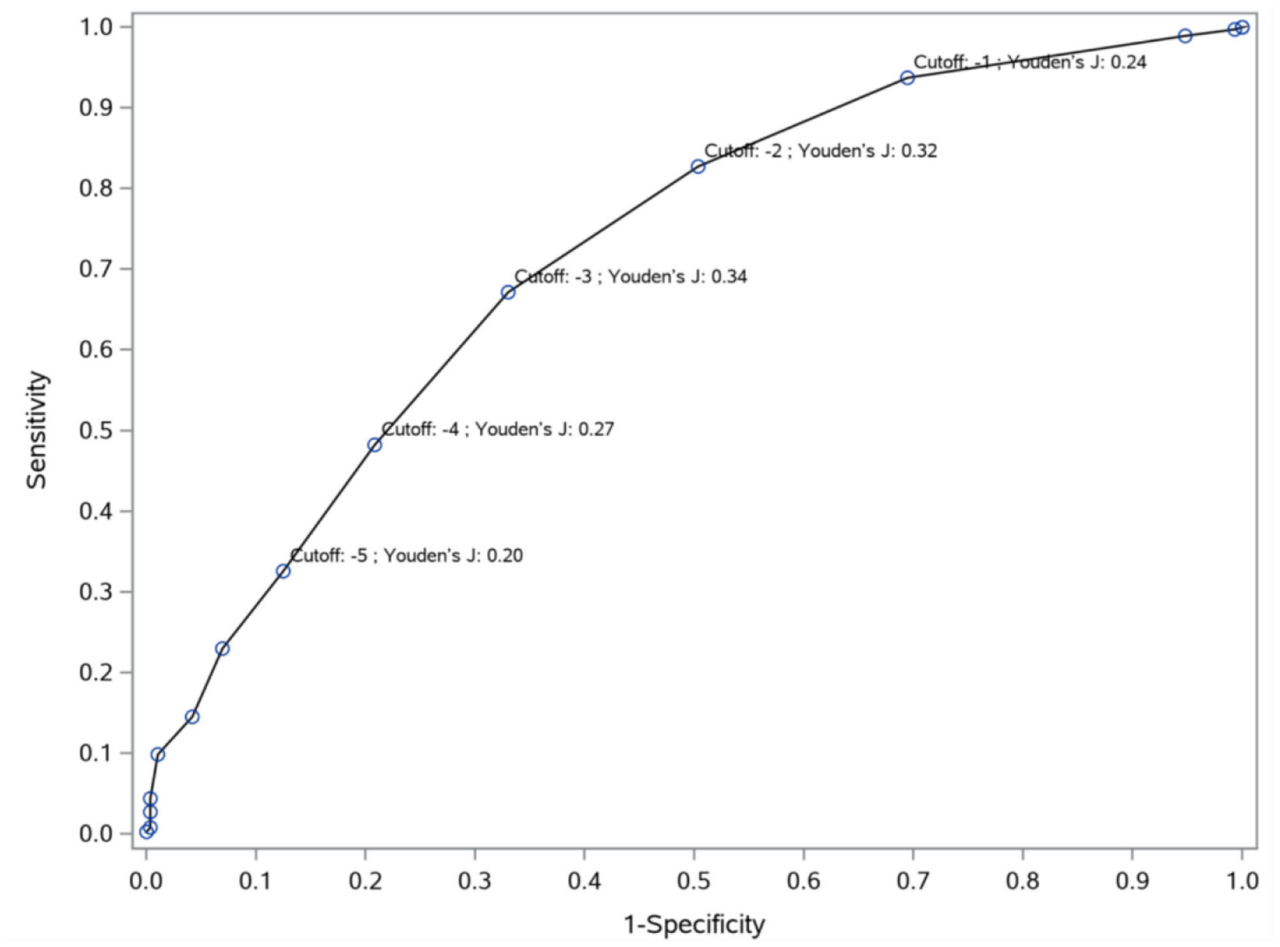
Cut-off values (Youden's indices) for predicting improvement from no clinical change to minimal improvement. To differentiate minimal improvement from no clinical change, Youden's J indices were computed at different cut-off points based on the PANSS-FSNS change. The sensitivity (vertical axis) and 1- specificity (horizontal axis) values for the differentiation of minimal improvement from no clinical change are depicted in the figure for various values of change from baseline in the PANSS-FSNS (labeled as “Cutoff”). Please note that the Youden's J index, which shows the efficiency of differentiation based on the combination of sensitivity and specificity, first increases then decreases with increasingly greater improvements (i.e., with greater negative values) as compared to baseline. The highest value of the Youden's J Index is reached at the cut-off value of −3 (i.e., at a 3 point reduction of symptom severity from baseline in the PANSS-FSNS), which identifies the optimal change value that maximizes sensitivity and specificity simultaneously.

The ROC curve indicated statistically robust predictive values of PANSS-FSNS changes, as the model fitted to our data (Model) was highly significantly different from the reference line (ROC1) (Figure 2). Based on the maximal Youden's index method (29) we identified a −3 point decrease from the PANSS-FSNS at baseline as the cut-off value most effectively differentiated between true positive and false positive classifications, i.e., minimally improved and unchanged statuses (Figure 3).

#### MCID_5_ as Odds Ratio

The odds ratio (OR) indicates the strength of association between the decrease in FSNS and the improved state based on CGI-I. For the −1.7 shift in FSNS obtained above as estimated MCID based on the Least Squares Mean Difference (LSMD), the OR is 1.86 (95% CI 1.62, 2.13; *p* < 0.05) favoring CGI-I = 3 vs. 4, The logistic regression analysis described above yielded an estimated OR of 2.07 (95% CI = 1.76 – 2.44; *p* < 0.05) for a 2-point decrease in the PANSS-FSNS factor score during the study. Thus, this analysis indicates that such an improvement (i.e., 2 points) in the PANSS-FSNS factor score more than doubles the likelihood of achieving minimal clinical improvement in the study.

## Discussion

Previous work has established the minimal improvement (CGI-I = 3) as being associated with a 27% decrease in the PANSS-FSNS for patients with predominantly negative symptoms of schizophrenia (20) which may still be an overestimation if the time effect of improvement is considered as well. The current analyses estimated the MCID with several methods by looking at it at its earliest occurrence.

It is important to note that the five different approaches that we adopted in the current investigation to characterize MCID complement each other and delineate the MCID from various vantage points. The distribution-free approaches characterize change over time in terms of the original units on the scale of interest (PANSS-FSNS), either as a baseline-end point difference associated with the minimal improvement on the CGI-I (score = 3) (MCID1) or the difference between unchanged and minimally improved PANSS-FSNS values (MCID2). The first one of the distribution-based methods that we adopted (MCID3) expresses the difference between unchanged and minimally improved PANSS-FSNS values (measured in the original scale units) in terms of standard deviation (statistical) units. The additional two distribution-based approaches employ logistic regression modeling in order to predict the minimally improved status on the basis of the PANSS-FSNS change over time. They express MCID either as measures of the ROC Curve (AUC, Youden's index in case of MCID4) or an OR (MCID5).

Applying these approaches, we confirmed that over time, more and more prominent symptom changes were needed to achieve minimal clinical improvement. In view of this finding, we conclude that the absolute minimum clinically meaningful difference should be considered at the earliest instance, since this approach provides the highest assay sensitivity to detect clinically important changes of symptom severity over time (i.e., it allows to capture the lowest symptom change threshold for minimal clinical improvement). We note that the PANSS-FSNS of the patients whose clinical status remained the same slightly decreased as well, although to a much smaller extent. This slight symptom reduction of the clinically not improving patients may be attributable to unspecific changes, such as the regression to the mean effect, a phenomenon often seen when applying strict inclusion criteria for the patients regarding their symptom severity (31). The presence of such unspecific changes, which evolve gradually with time, may make it more difficult to establish a clinically important difference at later times in a trial, thereby providing additional rationale for focusing on the earlier time points. Additionally, because improvement is rated against the initial baseline, anchoring changes of symptom severity to clinical improvement can be more and more difficult and accompanied by greater variation as the patients progress over time during the study.

Overall, applying various underlying concepts to calculate the MCID, different methods led to different estimates with respect to the PANSS-FSNS change that need to be considered as minimally clinically significant. Our estimates from the anchor-based analyses, ranging from 7.3 to 18.5% for patients with predominant negative symptoms of schizophrenia, were below the estimates reported from studies that relied on more heterogeneous patient populations and did not take the time effect into consideration. However, it is important to bear in mind that the distribution-based statistical approaches showed a marked separation (MCID_3_, i.e., statistical effect size in terms of standardized mean difference = 0.6); and highly significant predictive power for the PANSS-FSNS scale for differentiating Minimally Improved from Clinically Unchanged status in terms of ROC measures (MCID_4_; e.g., AUC = 0.7232) and the odds ratio (MCID_5_; OR = 2.07).

An important limitation of this investigation is that, although experienced raters met the training requirements and qualification criteria set forth before the rater training and administered the instruments, potential rating bias might have occurred. For example, the increasing PANSS-FSNS changes over time could be attributed to a possible oversight by comparing patients' clinical statuses to the previous visit instead of the baseline status (32). Furthermore, one could also argue that very early improvements might not have been real drug effects due to the two drugs' different pharmacokinetics and the onset of effect. Nevertheless, the MCID, by definition, is not about treatment-effect and could be driven by complex factors. Finally, since the CGI ratings were performed by the clinician, a further limitation of the study is that the minimally clinically important difference in the current investigation was evaluated from the clinician's perspective, not from the patient's. Patient-related outcomes were not assessed to determine the minimally clinically important change. However, we note that one study which examined patient- and clinician-rated CGI assessments simultaneously in patients with schizophrenia found only slight differences between the two approaches (18). Nonetheless, a specifically designed study is clearly needed to investigate this issue further. Further, an additional limitation derives from the use of only one particular scale. In this study we adopted one of the benchmark scales for negative symptoms of schizophrenia, the PANSS-FSNS. Assessing negative symptoms with different scales (such as the SANS, BNSS, CAINS, etc.) would yield different values as minimally important change, as scoring on these scales is different. Consequently, further research is needed to identify minimally clinically important changes on different negative symptom scales.

Negative symptoms, that are not secondary to positive ones, are known for being less responsive to antipsychotic therapy (33–35). Their presence often challenges the therapeutic strategy and makes clinicians switch the patients' medication. Our findings may help clinicians and drug developers have a more precise idea about improving predominant negative symptoms in clinical decision-making, or in terms of designing trials for patients with predominant negative symptoms.

## Conclusion

Applying different methods lead to different results, ranging between 7.3 and 18.5% improvement from baseline for the MCID at its earliest occurrence in patients with predominant negative symptoms of schizophrenia, suggesting even lower thresholds than previously thought.

## Data Availability Statement

The datasets presented in this article are not readily available because they are part of a bigger randomized clinical study dataset, and are owned by the company Gedeon Richter. The datasets have been provided to authors to perform the presented analyses only. While the full datasets can therefore not be shared, all statistical analyses and data outputs generated for the present study/publication can be requested by the authors.

## Ethics Statement

The clinical study protocol was approved by nine central and 37 local independent Ethics Committees in relation to the 66 sites that recruited at least one patient; the study was done in accordance with good clinical practice guidelines and the principles of the International Conference on Harmonization. All patients provided written informed consent. The patients/participants provided their written informed consent to participate in this study.

## Author Contributions

SL, PC, ÁB, IL, and GN developed the concept of the current investigation. Methodology contribution was provided by KA, PC, SL, and TAF. The statistical plan was outlined by PC and KA. KA conducted the statistical analyses under the supervision of PC. Software preparation was done by KA and PC. KA, PC, BS, and IL had a leading role in the preparation of the figures and table. Project administration was provided by ÁB, BS, IL, and GN. PC and KA interpreted the statistical outputs. Conceptual supervision for manuscript preparation and writing was provided SL, PC, and TAF. BS, IL, ÁB, and PC performed the writing. All authors contributed to the article and approved the submitted version.

## Conflict of Interest

PC, BS, KA, ÁB, IL, and GN reports personal fees from Gedeon Richter Plc., outside the submitted work. TAF reports grants and personal fees from Mitsubishi-Tanabe and from Shionogi, personal fees from MSD and from SONY, outside the submitted work. In addition, TAF has a patent 2020-548587 concerning smartphone CBT apps pending, and intellectual properties for Kokoro-app licensed to Mitsubishi-Tanabe. SL reports honoraria as a consultant/advisor and/or for lectures from Angelini, Böhringer Ingelheim, Geodon Richter, Janssen, Johnson & Johnson, Lundbeck, LTS Lohmann, MSD, Otsuka, Recordati, SanofiAventis, Sandoz, Sunovion, TEVA, Eisai, Rovi, and Medichem. GN and IL have issued patents for cariprazine. This study was sponsored by Gedeon Richter Plc. Gedeon Richter was involved in the study design, collection (via contracted clinical investigator sites), analysis, and interpretation of data and decided to submit it for publication.

## Publisher's Note

All claims expressed in this article are solely those of the authors and do not necessarily represent those of their affiliated organizations, or those of the publisher, the editors and the reviewers. Any product that may be evaluated in this article, or claim that may be made by its manufacturer, is not guaranteed or endorsed by the publisher.
